# AI Triage in Primary Care: Building Safer and More Equitable Real-World Evidence

**DOI:** 10.2196/88396

**Published:** 2026-03-04

**Authors:** Aymn Alamoudi, Evangelos Kontopantelis, Salwa S Zghebi, Benjamin Brown

**Affiliations:** 1 Division of Population Health, Health Services Research and Primary Care, School of Health Sciences, Faculty of Biology, Medicine and Health University of Manchester Manchester, England United Kingdom; 2 Department of Public Health, School of Nursing and Health Sciences Jazan University Jazan, Jazan Region Saudi Arabia; 3 Division of Informatics, Imaging and Data Sciences, School of Health Sciences, Faculty of Biology, Medicine and Health University of Manchester Manchester, England United Kingdom; 4 Division of Family Medicine Yong Loo Lin School of Medicine National University of Singapore Singapore Singapore

**Keywords:** artificial intelligence, AI, triage, primary care, general practice, patient safety, health equity, real-world evidence, clinical decision support

## Abstract

Artificial intelligence triage in general practice is developing rapidly within the primary care digital transformation, promising efficiency gains and safety standardization in overwhelmed primary care systems. However, current evidence is drawn from retrospective validations, emergency settings, or vignettes, with scant evaluation of real-world outcomes and almost no equity-stratified safety data, despite known disparities across age, ethnicity, language, and deprivation. From a sociotechnical standpoint, which considers the fit between people, tasks, technology, and organizational context, risks arise not only from algorithmic bias and undertriage but also from human factors, workflow misalignment, governance gaps, and inadequate postdeployment monitoring. We argue that ensuring artificial intelligence triage is safe and equitable requires real-world evaluations in primary care settings, equity-focused performance reporting using theoretically informed frameworks, and rigorous postmarket surveillance. Without these, deployment may widen existing health inequalities rather than moderate them.

## Introduction

**Figure 1:**
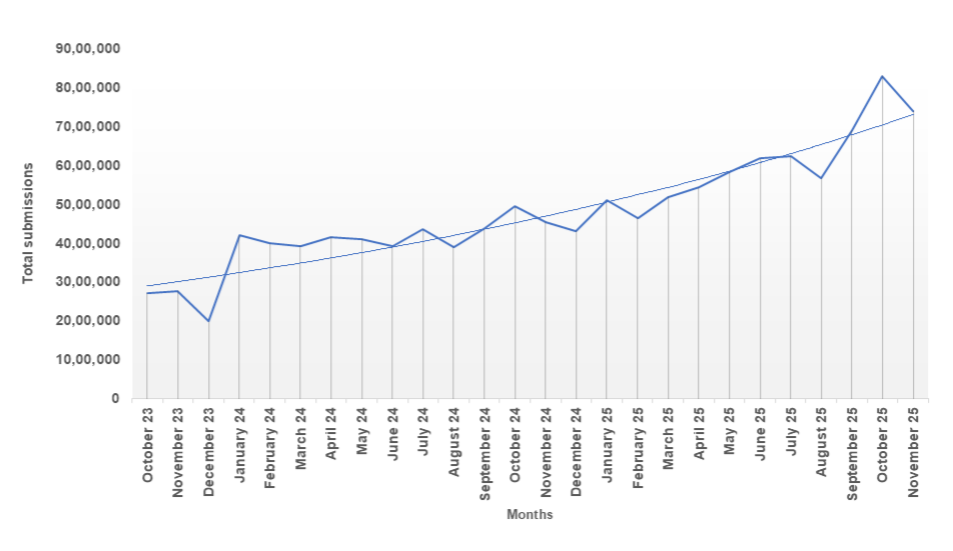
Growth in monthly online consultation submissions in England. Data source: National Health Service England release [6]. Information from NHS England, licenced under the current version of the Open Government Licence.

Globally, primary care faces sustained growth in demand, increased patient complexity, and a workforce whose full-time equivalent growth has not matched demand, resulting in persistent access pressures and delays [1,2]. The COVID-19 pandemic accelerated the adoption of remote and digital access, including online consultations and reinforced strategic commitments to “digital front door” models within health systems, such as the National Health Service [3-5]. Online consultation submissions in England rose from approximately 2.7 million in October 2023 to a peak of 8.3 million in October 2025, as seen in Figure 1, highlighting the rapid and sustained growth of digital entry points into general practice (GP) [6]. Evidence also suggests that digital access is not equity neutral. A systematic review of inequalities in remote GP consultations found differential use by sociodemographic characteristics, with internet-based consultations more frequently used by younger, more affluent, and more educated groups, and noted that the impact of these inequalities on clinical outcomes remains uncertain [7].

In this context, artificial intelligence (AI)–enabled triage combines structured questions, red-flag pathways, and machine learning (ML) risk stratification with electronic health record (EHR) integration and clinician oversight to route patients more efficiently and potentially improve safety [8-10]. In this viewpoint, we distinguish between 3 related but conceptually distinct system types: symptom checkers, clinical decision support systems (CDSSs), and AI triage. Symptom checkers are patient-facing digital tools that provide health advice or triage recommendations directly to users, often without clinician oversight, and have been widely evaluated in consumer and emergency contexts [8]. CDSSs are clinician-facing tools embedded within clinical workflows or EHRs that support decision-making through alerts, risk scores, or guideline-based recommendations [9,10]. AI-enabled triage refers to digital systems that collect patient-reported information and generate urgency or routing recommendations (eg, self-care, routine review, urgent GP assessment, or emergency referral), with or without clinician oversight [11]. These systems may be embedded within online consultation platforms, patient-facing symptom checkers, or CDSS. Importantly, not all online consultation systems are AI enabled and not all AI-enabled triage systems function as stand-alone symptom checkers.

This viewpoint advances 3 linked arguments. First, we argue that triage in primary care is a safety-critical and equity-sensitive function, such that errors or delays can produce serious harm and unequal outcomes. Second, we show that the current evidence base for AI-supported triage is dominated by emergency department (ED), vignette, and retrospective studies, with little real-world or equity-stratified evaluation in GP. Third, we argue that AI triage operates as a sociotechnical system shaped by human behavior, workflows, and governance, meaning that algorithmic accuracy alone cannot guarantee safety or fairness. This viewpoint aims to outline a practical agenda for evaluating and governing AI-enabled triage in GP that integrates real-world safety outcomes, equity-stratified performance reporting, and sociotechnical implementation and surveillance. The intended audience includes GP clinicians and practice leaders, digital health and AI developers, evaluators and implementation scientists, and policymakers and regulators responsible for deployment and monitoring. Our contribution is to consolidate a practical, real-world evaluation and governance agenda for AI triage in GP that integrates sociotechnical safety (workflow and human factors), equity-stratified performance reporting (including an example of fairness), and postdeployment surveillance.

### Triage in Primary Care Is Safety Critical and Equity Sensitive

In health care, triage refers to the systematic process of assessing patient urgency and risk to determine the appropriate level, timing, and pathway of care [12,13]. In primary care, triage does not establish a diagnosis but prioritizes patients for self-care, routine review, urgent GP assessment, or emergency referral based on presenting symptoms, clinical risk, and service capacity [14,15]. This function is safety critical because misclassification can lead to delayed diagnosis, inappropriate self-management, or unnecessary escalation [8-10]. AI-enabled triage promises standardization and auditability but introduces novel patient-safety risks, such as automation bias, algorithmic mistriage, and digital exclusion, particularly for socially disadvantaged groups [8,16,17]. Moreover, model performance (eg, sensitivity, specificity, calibration, and error rates) may vary by age, ethnicity, language, or limited digital access, unless these dimensions are intentionally tested and monitored [18-22].

The absence of equity evaluation may pose a significant risk. If models are calibrated primarily on majority language, younger, or White-majority cohorts, AI triage may systematically de-escalate or deprioritize patients whose symptom descriptions diverge due to cultural or linguistic factors. Coupled with cognitive and automation biases in human users, the most vulnerable groups risk unsafe disposition, such as self-care advice when urgent assessment is indicated.

The “equity blind spot” in AI triage is not merely a technical glitch; it reflects broader systemic oversight. To operationalize safe, equitable AI, embedding framework-informed stratification, for example, PROGRESS-Plus, fairness metrics, and multidimensional performance reporting into every stage of model development, validation, and deployment is needed. Without this, AI triage may reinforce and even amplify existing health care disparities if deployed without adequate safeguards.

## What the Evidence Shows and What It Misses

### What Current Studies Show

Controlled evaluations of AI triage report high technical performance, with area under the receiver operating characteristic (singular) curve values typically between 0.82 and 0.94 and sensitivities often exceeding 0.75. However, these studies are predominantly retrospective, vignette based, or conducted in EDs and hospital settings. They rarely reflect routine workflows in GP [23-26].

Recently, Abualruz et al [12] reviewed 22 studies on AI-supported triage, most of which were carried out in emergency, acute, or hospital-based settings. Only a small subset examined outpatient or primary-care use. As a result, the current literature tells us little about how AI triage performs in everyday GP or how it affects patient safety in real workflows. Table 1 presents the distribution of published AI-supported triage studies by clinical setting.

**Table 1:** Distribution of published artificial intelligence–supported triage studies by clinical setting (N=22).

Clinical setting	Study type	Studies, n (%)
Emergency department or hospital	Real patient data	19 (86)
Primary care	Clinical vignettes or qualitative studies	3 (14)
Primary care	Real patient data	0 (0)

Most studies have been conducted in emergency or hospital settings using real patient data (19/22, 86%). In total, 14% (3/22) of the studies relied on clinical vignettes or qualitative interviews, and none (0/22, 0%) evaluated AI-supported triage using real-world patient data in routine GP.

### Why This Evidence Is Not Sufficient for Safe and Equitable GP Deployment

Equity reporting is also sparse. Few studies disaggregate performance by age, ethnicity, language, or socioeconomic status [27-30]. Intersectional analyses, for example, age×ethnicity or ethnicity×deprivation, are almost absent. Subgroup calibration, false-negative rates, and false-positive rates (FPRs) are rarely reported.

Study design further limits interpretability. Vignette-based and retrospective analyses do not capture real-world pressures, such as workload variation, free-text symptom input, clinician overrides, or case-mix drift [31]. Prospective designs, such as controlled interrupted time series or cluster-randomized trials, are almost never used in GP settings [1,3]. Without these designs, safety effects cannot be attributed reliably to AI deployment.

Postdeployment monitoring is also underdeveloped (Table 2). Few studies report ongoing calibration checks, subgroup performance dashboards, or systematic incident reporting aligned to the World Health Organization (WHO) International Classification for Patient Safety [32,33]. As a result, health systems lack visibility into how AI triage safety changes over time.

**Table 2:** What the evidence on artificial intelligence triage shows and what it misses.

Domain	What studies typically show	What is usually missing
Accuracy	High area under the receiver operating characteristic curve (≈0.82-0.94) and reasonable sensitivity in retrospective and vignette-based studies	Performance under real general practitioner workload, with free-text input, comorbidity, and clinician override
Setting	Predominantly emergency departments, acute care, or simulated cases	Routine general practice, community clinics, and longitudinal follow-up
Safety outcomes	Agreement with clinicians or reference standards	Delayed diagnosis, avoidable emergency use, or patient harm
Equity	Rare or absent subgroup reporting	Performance by age, ethnicity, language, deprivation, or intersectional groups
Monitoring	One-off validation at model development	Postdeployment drift, subgroup miscalibration, and incident tracking

Consistent with this, an ED-focused scoping review found limited demographic breakdowns and no multidimensional analyses, leaving equity implications unclear [27]. Within UK GP settings, stratified data on undertriage or performance by deprivation or ethnicity are rare [16,29], and subgroup calibration metrics or true-positive rate (TPR) and FPR reporting are notably absent. A recent international review found variable triage accuracy, poor calibration reporting, and limited deployment-level evaluation, reinforcing that the evidence gap is global [34].

## Why AI Triage Is a Sociotechnical System

### Overview

AI-enabled triage systems are not isolated algorithms; they operate within complex care delivery systems where human factors, workflows, and trust dynamics profoundly shape safety. A sociotechnical system perspective, exemplified by the systems engineering initiative for patient safety framework, analyzes how people, tasks, tools, and organizational structures interact to influence patient safety.

By contrast, implementation frameworks focus on organizational readiness, technology adoption, and sustainability [35,36]. Together, these complementary approaches emphasize that deploying an accurate ML model alone does not guarantee safe outcomes; safety depends on both workflow integration and organizational adoption.

Human factors and trust are central. Health care professionals and patients must interpret AI-generated recommendations within their cognitive, ethical, and emotional contexts. A recent qualitative work on AI-based triage in Swedish primary care underlines how trust emerges from lived experience, transparency, and perceived reliability. Both patients’ and professionals’ trust is contingent on real-world usability and clear decision roles and not just model accuracy [31].

Similar issues are emerging in teledentistry and dental triage, where AI-enabled chatbots and triage systems are used to prioritize pain, infection, trauma, or urgent referral pathways [37]. Early work includes prototype “intelligent dental triage systems” and evaluations of AI chatbots for dental queries, but the same core risks apply: safety-critical undertriage, unequal performance for patients with language barriers or limited digital access, and workflow integration challenges in busy dental practices [38]. AI tools in dental assessment and smile analysis, such as Dynasmile, a video-based AI smile analysis platform in orthodontics, illustrate the expanding role of AI beyond workflow triage into diagnostic and aesthetic decision support in oral care [39].

This cross-domain comparison reinforces our central claim: AI triage should be evaluated as a sociotechnical intervention with equity-stratified safety reporting and postdeployment monitoring, regardless of clinical specialty.

### Explainable AI to Support Calibrated Trust and Reduce Automation Bias

In safety-critical triage, explanations should aim to support calibrated trust not persuasion. Evidence from clinical decision-support research suggests that well-designed explanations can improve clinician understanding and trust calibration, while poorly designed explanations can increase overreliance and automation bias [40,41].

In practice, explainable AI for GP triage should be workflow integrated and low burden, including the following: (1) a short list of the main drivers for escalation (eg, red-flag symptoms and abnormal risk profile), (2) uncertainty indicators or confidence bands, (3) an “override required” prompt for high-risk edge cases, and (4) safety-netting text that is consistent with the triage rationale. Explanation stability is also important; near-identical inputs should not produce inconsistent rationales, as this undermines trust and may increase unsafe deference [40,42].

These requirements for explanation design reinforce the importance of sociotechnical fit described subsequently.

### What Real-World Deployments Show

AI triage does not operate as a stand-alone algorithm. In GP, it is embedded in symptom checkers, online consultations, patient apps, and EHR-based decision-support tools. These systems influence how patients describe symptoms, how clinicians prioritize work, and how care is delivered.

Evidence from multiple countries shows potential efficiency gains. In Iceland, an ML triage model for respiratory symptoms improved previsit risk stratification in community clinics [43]. Similarly, Brazil’s primary care referral triage system demonstrated improved appropriateness of specialist referrals [44]. Studies from Sweden and Italy reported improved workflow transparency but persistent concerns about trust, usability, and clinician acceptance [31,33].

However, most deployments remain early stage, small scale, or limited to specific pathways. Systematic reviews of symptom checkers across Europe, the United States, Spain, Canada, and Asia report wide variability in triage accuracy and frequent mismatches between algorithmic and clinician assessments, particularly for complex multimorbidity and non–native-language users [8,14,34,45].

### Why Workflow, Trust, and Integration Matter

Qualitative evidence highlights that sociotechnical fit is critical. Steerling et al [31] found that both health care professionals and patients require alignment with clinical judgment, transparency, and oversight before trusting AI-based triage. Similarly, Siira et al [46] identified 3 interacting barriers in Swedish primary care: (1) professional skepticism or resistance and trust, (2) organizational readiness and digital maturity, and (3) technical limitations and poor EHR integration. Successful sites mitigated these by hands-on leadership and staff training, “superuser” networks, and iterative codevelopment with vendors. Even where efficiency gains were perceived, unresolved integration gaps and complex case-mix sustained workload and safety concerns.

Evidence from the UK primary care e-visits (14 practices; 16 staff, 24 patients; 2020-2021) identified 7 concrete AI use cases—workflow routing, directing, prioritization, postsubmission adaptive questioning, writing assistance, self-help information, and autobooking—and found acceptability hinged on clinical oversight, timely responses, and ongoing evaluation. Perceived upsides were workload relief and faster help, while risks were depersonalization and mistriage, if poorly implemented [47].

### What This Means for Safety

These findings show that AI triage safety depends on how tools interact with people, workflows, and organizational routines. This aligns with sociotechnical safety theory, particularly the systems engineering initiative for patient safety framework, which emphasizes the fit between tasks, technology, and organizational context [32,48,49].

AI also offers enhanced structured data capture, natural language processing (NLP)–enabled symptom interpretation, EHR-integrated safety netting, and auditable decision trails [50]. NLP-enabled symptom interpretation can, for example, recognize “feeling pressure in chest” as equivalent to angina, supporting safer triage for patients who do not use standard terminology [26,45]. However, these benefits are offset by risks. Algorithmic mistriage, automation bias, and poor integration can delay escalation or overload urgent pathways [8,13].

Without governance mechanisms, such as version control, audit logs, safety dashboards, and periodic revalidation, performance may degrade over time as populations, language, and risk profiles change [32]. Therefore, sociotechnical alignment is not optional; it appears essential for safe AI-enabled triage.

## The Equity Blind Spot

Although AI-enabled triage systems are promoted as equitable tools for managing primary care demand, the current evidence reveals a persistent equity blind spot, driven by underrepresentation, limited fairness measurement, and neglect of equity-focused monitoring [51].

Equity in digital health demands more than equal treatment; it requires fair opportunity to achieve safe, good outcomes, especially when baseline disadvantages exist. Equity reporting should use structured tools. The PROGRESS-Plus framework, developed by the Cochrane-Campbell equity methods group, was designed to systematically identify and report on equity-relevant factors in health research [17]. It extends the original PROGRESS (place of residence, race and ethnicity, occupation, gender, religion, education, socioeconomic status, and social capital) acronym with “plus” dimensions, including age, disability, and language [17]. The framework was created to help researchers illuminate disparities that might otherwise be masked in aggregated data and has since been widely applied in clinical trials, systematic reviews, and digital health evaluations.

Alternative frameworks, such as the health equity impact assessment tool, as seen in Textbox 1, are used prospectively to anticipate equity impacts before interventions are deployed [52]. The SIITHIA (Strengthening the Integration of Intersectionality Theory in Health Inequality Analysis) checklist provides structured criteria for identifying inequities in digital health [53]. More recently, the digital health equity framework extends this approach to digital interventions and multidimensional analyses [54]. In practice, these can be operationalized by setting thresholds (eg, sensitivity gaps ≤5% between groups), with breaches triggering model review and corrective action. Complementing this, algorithmic fairness metrics, such as equal opportunity (equal TPRs), equalized odds (matching both TPR and FPR), and calibration integrity, are critical for measuring subgroup performance and detecting systematic bias. Without these frameworks, inequities may go undetected beneath aggregated performance.

**Frameworks for evaluating safety and equity in artificial intelligence triage.**

**Safety and sociotechnical performance**

- Systems engineering initiative for patient safety describes how people, tasks, tools, and organizational context interact to shape patient safety [48].
- World Health Organization International Classification for Patient Safety provides a standardized taxonomy for reporting and classifying safety incidents [32,49,55].

**Implementation and adoption**

- Consolidated Framework for Implementation Research assesses organizational readiness and barriers to and facilitators of implementation.
- Nonadoption, abandonment, scale-up, spread, and sustainability [35,36] framework evaluates the complexity and long-term viability of digital health technologies.
- Reach, effectiveness, adoption, implementation, and maintenance framework evaluates long-term reach, effectiveness, adoption, and maintenance [56].
- Human, organization, and technology-fit framework examines alignment between human, organizational, and technical factors [57].

**Equity and fairness**

- PROGRESS-Plus identifies social stratifiers, such as age, ethnicity, language, deprivation, and disability [17].
- Health Equity Impact Assessment tool evaluates potential equity impacts before deployment [52].
- The SIITHIA (Strengthening the Integration of Intersectionality Theory in Health Inequality Analysis) checklist and the digital health equity framework support intersectional and digital-specific equity analysis [53,54].

The frameworks presented in Textbox 1 allow AI triage to be evaluated across safety, implementation, and equity dimensions. Combining them enables a more comprehensive assessment than any single lens can provide.

Textbox 2 provides an illustrative example of how to report equal opportunity (true positive rate; sensitivity) across intersectional PROGRESS-Plus strata.

**Illustrative example: reporting equal opportunity across intersectional strata.**

- Equal opportunity requires similar true positive rates (TPRs; sensitivity) across groups for individuals who truly need urgent care.
- Step 1 includes defining the safety-critical outcome (Y=1), for example, “urgent same-day clinical assessment required” based on a reference standard (eg, clinician adjudication, emergency department attendance within 24 to 48 hours, or diagnosis of a time-critical condition).
- Step 2 involves computing TPR within each subgroup.
- TPR (sensitivity) = true positive (TP) / (TP + false negative [FN])
- Step 3 involves reporting TPR across PROGRESS-Plus strata and intersectional strata, for example, age group×ethnicity or ethnicity×deprivation quintile. An example of reporting is as follows:
- White, least deprived: TP=180; FN=20 → TPR=0.90
- White, most deprived: TP=150; FN=30 → TPR=0.83
- Minority ethnicity, least deprived: TP=70; FN=20 → TPR=0.78
- Minority ethnicity, most deprived: TP=55; FN=25 → TPR=0.69
- Step 3a involves quantifying uncertainty in subgroup estimates. For each subgroup, TPRs should be reported with measures of uncertainty (eg, 95% CIs or standard errors), particularly where subgroup sample sizes are small. This enables assessment of whether observed differences are robust or compatible with random variation.
- Step 4 summarizes the disparity. This approach makes equity risks visible that would be hidden in overall performance metrics.
- Absolute gap (maximum TPR – minimum TPR) = 0.90 – 0.69 = 0.21
- Flag threshold: investigate if the gap is greater than 0.05 or if any subgroup TPR is less than 0.80
- These figures are illustrative. In practice, equity assessments should consider statistical uncertainty (eg, CI overlap and subgroup sample size) alongside point estimates. Empirical data on equity-stratified artificial intelligence triage performance in primary care remain limited.

Several dimensions of bias have been documented or are anticipated in an AI-driven triage system, as mentioned subsequently.

The first dimension is age. Older adults are frequently underrepresented in development datasets, increasing the risk of atypical presentations or those with limited digital literacy [58,59].

The second dimension is language and ethnicity. NLP models are extremely sensitive to linguistic variation, dialects, multilingual input, or limited English proficiency. However, model evaluations rarely account for these, threatening safe triage in diverse populations [60].

Furthermore, broader AI research (outside primary care) shows that racial and ethnic biases in algorithmic systems persist [61]. For instance, algorithms that relied on health care cost as a proxy for illness systematically undertriaged Black patients due to unequal access-driven cost differences [60]. Similarly, AI in imaging frequently underdiagnoses emergent pathology in marginalized groups. Black women have shown significantly higher underdiagnosis rates in medical imaging models.

Telephone or digital triage evaluations suggest that low-income individuals, ethnic minority groups, and displaced patients experience worse outcomes, though quantitative data remain sparse [62].

Single-axis analysis (age or ethnicity) is insufficient. Intersecting vulnerabilities (eg, older adults from minority ethnic backgrounds with language barriers) can compound risk and increase mistriage. However, only a limited number of studies disaggregate safety performance by intersectional subgroups, leaving some of the most disadvantaged populations effectively invisible in assessments. Such analyses often lack power; therefore, findings should be treated as exploratory unless supported by large, multisite cohorts.

## What Must Change: a Research and Policy Agenda

**Figure 2:**
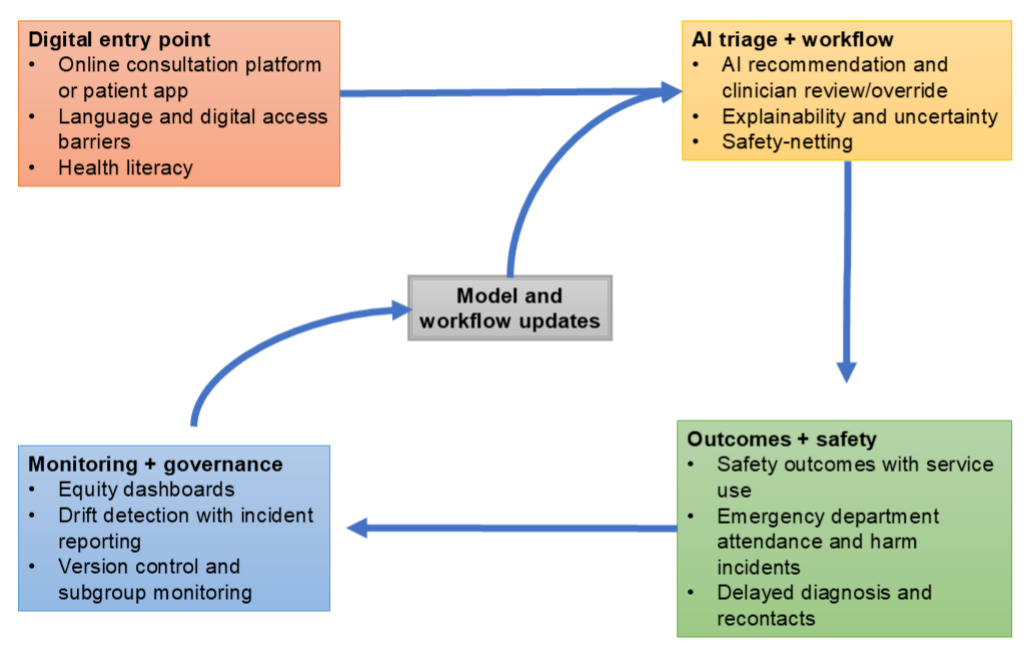
Conceptual loop for evaluating and governing artificial intelligence (AI)–enabled triage in general practice as a sociotechnical intervention.

In this viewpoint paper, we argue that AI triage stands at a crossroad; it has the potential to improve safety and access in primary care but requires real-world evaluation, equity-focused monitoring, and sociotechnical governance. Figure 2 summarizes the proposed real-world evaluation and governance loop for AI triage in GP. Digital entry points feed into AI-supported triage and clinician workflow, producing safety and service-use outcomes that inform monitoring and governance (including equity dashboards, drift detection, and incident reporting). Governance outputs drive model and workflow updates, enabling continuous improvement.

To shift AI triage from a hypothetical promise to an equitable, safe reality in GP and primary care settings, we propose 5 interrelated priorities, as mentioned subsequently.

The first priority is real-world evaluations in primary care (prospective or retrospective). Current evidence is dominated by vignette experiments or ED contexts. Prospective, real-world evaluations—such as controlled interrupted time series or (cluster) randomized controlled trials (RCTs)—that assess patient safety outcomes (eg, delayed diagnoses and avoidable emergency use), workflow effects, and override behaviors in GP practice are urgently needed. Where randomization is infeasible, controlled interrupted time series with matched controls and prespecified safety outcomes can provide strong quasi-experimental evidence.

The second priority is equity-stratified performance reporting. AI triage systems should be evaluated through the equity lenses, such as PROGRESS-Plus and other fairness metrics. This means disaggregating performance (TPR, FPR, and calibration) by age, ethnicity, language, deprivation, and their multidimensional aspects to identify and mitigate differential risks. Without such reporting, disparities will remain hidden.

The third priority is causal evaluation designs. Observational signals are helpful, but causality demands rigorous designs. Interrupted time series around AI deployment and RCTs, where feasible (to attribute safety effects more definitively to AI interventions), and propensity score methods, instrumental variables, or target trial emulation, where RCTs are infeasible, should be conducted.

The fourth priority is postmarket surveillance and governance infrastructure. Effective governance is not optional. Organizations should adopt frameworks such as people, process, technology, and operations to ensure structured oversight across personnel, process, technology, and operations. AI triage requires continuous monitoring, with statistically principled detection of drift, subgroup miscalibration, and emerging hazards [63].

The fifth priority is human-AI collaboration and implementation research. Research should shift from algorithm-centric evaluation to sociotechnical integration. Studies should examine how clinicians interpret, override, or trust AI suggestions; how AI supports (rather than disrupts) workflow; and how organizational culture shapes safe AI adoption. Mixed methods research combining qualitative insights with quantitative safety metrics will be critical.

Prospective evaluations in GP are challenging but feasible. Cluster-randomized trials and interrupted time series require careful handling of contamination between clinicians, cointerventions during rollout, seasonal and demand shocks, and variation in practice digital maturity. Outcome measurement also depends on data linkage (eg, EHR, urgent care, ED attendance, and diagnostic follow-up), and governance processes can slow implementation. Although such designs remain underused for AI-enabled triage, they are well-established in evaluating complex service interventions in primary care and are methodologically appropriate for this context [64,65].

## Conclusions

AI triage offers potential for improving primary care efficiency, safety, and consistency, but current evidence leaves critical gaps. Without intersectional, real-world safety evaluations, implementation is not just uncertain; it may be ethically risky and may inadvertently magnify existing health inequities. Over the coming years, this field must commit to responsible, equity-focused, system-aware evidence generation. That means embedding AI evaluation within the disordered realities of practice, governance mechanisms that ensure fairness and transparency, and human-AI systems that augment care rather than add workload. Operationally, this requires three commitments: (1) prospective, real-world evaluations in GP; (2) equity-stratified performance reporting guided by frameworks, such as PROGRESS-Plus; and (3) rigorous postmarket surveillance with drift and subgroup monitoring and WHO International Classification for Patient Safety–aligned incident reporting. Without these, deployment risks amplify inequities rather than reducing them. With these commitments, AI triage can better deliver on its potential of safer, more equitable primary care.

## Abbreviations

AI: artificial intelligence
CDSS: clinical decision support system
ED: emergency department
EHR: electronic health record
FPR: false-positive rate
GP: general practice
ML: machine learning
NLP: natural language processing
RCT: randomized controlled trial
SIITHIA: Strengthening the Integration of Intersectionality Theory in Health Inequality Analysis
TPR: true-positive rate
WHO: World Health Organization
